# Supplementary material for: Peroxisomal β-oxidation enzyme, DECR2, regulates lipid metabolism and promotes treatment resistance in advanced prostate cancer
Source: Br J Cancer. 2024 Jan 12;130(5):741–54. doi: 10.1038/s41416-023-02557-8 (PMC10912652; doi:10.1038/s41416-023-02557-8)
Supplement: Supplementary file 1 — Supplementary Information [file 41416_2023_2557_MOESM1_ESM.pdf]

SUPPLEMENTARY INFORMATION

Supplementary Figure 1 (a) Heatmap of KEGG peroxisome pathway genes in the Taylor cohort. **(b)** C42B and 22Rv1 prostate cancer cell lines treated with TDZ (2.5 μM) were assessed for cell migration using a transwell migration assay. **(c)** C42B, V16D and MR49F prostate cancer cells were treated with TDZ for 48 h and assessed for apoptotic and dead cells via flow cytometry. Data presented as percentage of cells in each live, apoptotic, or dead state per sample. **(d)** Histogram displaying DECR2 mutation and copy-number alteration frequency across 9 prostate cancer genomic datasets (left), and across 3 prostate cancer subtypes (right). **(e)** Quantification of the proliferative marker Ki67 in vehicle (VEH) or TDZ-treated (10 μM and 20 μM) patient-derived explants (PDEs). **(f)** Representative p63/AMACR and DECR2 IHC staining of intra-tissue benign and malignant regions. Scale bar, 50 μm. **(g)** Immunocytochemistry staining of LNCaP and 22Rv1 cells to determine subcellular localisation of DECR2. DAPI: nuclei; Alexa Fluor 488 secondary antibody: DECR2; Alexa Fluor 594 secondary antibody: PMP70 (Peroxisome), scale bar = 10 μm. **(h)** Cell viability of overexpression hDECR2 cells versus hControl LNCaP cells after treatment with varying doses of TDZ. **(i)** Viability of V16D cells subjected to siRNA-mediated DECR2 knockdown with or without TDZ treatment. Abundance of acyl-carnitine species in **(j)** DECR2 knockdown V16D prostate cancer cells supplemented with 100μM DHA, and **(k)** in TDZ-treated (7.5μM and 10μM) V16D prostate cancer cells. All cell line data are representative of at least 2 independent experiments and presented as mean ± s.e.m of triplicate wells. Statistical analyses were performed using ordinary one-way or two-way ANOVA: *$p < 0.05$, **$p < 0.01$, ***$p < 0.001$ and ****$p < 0.0001$.

Supplementary Figure 2 (a) Cell death of androgen-dependent LNCaP, castrate-resistant 22Rv1 and V16D, and enzalutamide-resistant MR49F prostate cancer cell lines subjected to siRNA-mediated DECR2 knockdown. **(b)** Cell viability and cell death of non-malignant prostate PNT1 cells. Cell viability and cell death were measured using Trypan blue exclusion and manual cell counting 96 h post DECR2 knockdown. Percentages are represented relative to the control siRNA. Cell viability of **(c)** LNCaP and **(d)** 22Rv1 cells with stable/inducible shRNA control (shControl) or DECR2 knockdown (shDECR2). Colony formation of **(e)** LNCaP cells with stable/inducible shRNA control and **(f)** 22Rv1 cells with stable/inducible shRNA control and DECR2 knockdown. **(g)** C42B and 22Rv1 prostate cancer cell lines subjected to siRNA-mediated DECR2 knockdown were assessed for cell migration using a transwell migration assay. Scale bar, 100μm. **(h)** *left:* Tumour weight and lung luminescence readings following DECR2 knockdown in mice (shDECR2+dox $n = 11$, shDECR2-dox $n = 10$). *middle:* Correlation of tumour weight data and luminescence intensity from DECR2 knockdown mice. *right:*

Tumour weight includes data from mice with sufficient sized tumours for analysis (shDECR2+dox *n* = 5, shDECR2-dox *n* = 8). **(i)** Tumour growth and lung luminescence readings of DECR2 overexpression mice (*n* = 10 per group, including mice with non-detectable tumours). All cell line data are representative of at least 2 independent experiments and presented as mean ± s.e.m of triplicate wells. Statistical analyses were performed using ordinary two-way ANOVA, or two-tailed student's t-test: ns = non-significant, *$p < 0.05$, **$p < 0.01$, ***$p < 0.001$.

Supplementary Figure 3 (a) Significantly enriched MSigDB Hallmark terms among differentially expressed genes. Quantitative PCR (qPCR) of cell cycle-related genes in **(b)** DECR2 knockdown V16D and MR49F cells, and **(c)** dox-inducible shDECR2 knockdown cells and LNCaP overexpression hDECR2 cells. **(d)** Cell cycle distribution of LNCaP cells with stable overexpression of DECR2, treated with ribociclib (Rib; 0.1 µM and 0.25 µM). **(e)** Viability of LNCaP cells with stable overexpression of DECR2, treated with ribociclib. **(f)** Top 30 transcription factors (TFs) that were enriched in our list of top upregulated differentially expressed genes ($p < 0.01$, log2 fold-change ≥ 1) using the MeanRank method in ChEA3 [26]. TFs are ranked from 1 to 30 in ascending order (from left to right), bubble size indicates the number of genes corresponding to the TF targets. All *in vitro* data are representative of at least 2 independent experiments and presented as mean ± s.e.m of triplicate wells. Statistical analyses were performed using ordinary one-way or two-way ANOVA. *$p < 0.05$, **$p < 0.01$, ***$p < 0.001$ and ****$p < 0.0001$.

Supplementary Figure 4 (a) V16D and MR49F prostate cancer cells subjected to siRNA-mediated DECR2 knockdown were assessed for neutral lipid content via flow cytometry. **(b)** V16D and MR49F prostate cancer cells were treated with TDZ for 48 h and assessed for neutral lipid content via flow cytometry. Total lipid abundance in **(c)** LNCaP, V16D and MR49F cells, and in **(d)** DECR2 overexpressing LNCaP cells. **(e)** Quantitative (left panel) and relative (right panel) abundance of each lipid class in LNCaP, V16D and MR49F cells. **(f)** Quantitative (left) and relative (right) abundance of each lipid class in DECR2 overexpressing LNCaP cells. Statistical analyses were performed using ordinary two-way ANOVA, or two-tailed student's t-test. *$p < 0.05$, **$p < 0.01$, ***$p < 0.001$ and ****$p < 0.0001$.

Supplementary Figure 5 (a) GSEA of peroxisomal Hallmark and KEGG proteins shows positive correlation with acquired resistance to apalutamide. **(b)** V16D and MR49F colony formation was evaluated in cells subjected to siRNA-mediated DECR2 knockdown, with or without enzalutamide, ENZ (1 or 10 µM) treatment. **(c)** 22Rv1 and MR49F colony formation was evaluated in cells treated with TDZ (1 and 2.5 µM) and/or ENZ (10 µM). **(d)** Cell cycle

distribution of LNCaP cells with stable overexpression of DECR2 cultured in DCC media. Statistical analysis was performed using ordinary two-way ANOVA. *$p < 0.05$, **$p < 0.01$, ***$p < 0.001$ and ****$p < 0.0001$.

Supplementary Data 1 Analysed RNA sequencing data and sample metadata

Supplementary Data 2 Analysed lipidomic data and sample metadata

Supplementary Table 1 The clinicopathologic features of prostate cancer patients included in this study

Supplementary Table 2 List of primary antibodies

Supplementary Table 3 Primer sequences qPCR

Supplementary Table 4 shRNA and hRNA sequences

Supplementary Figure 1

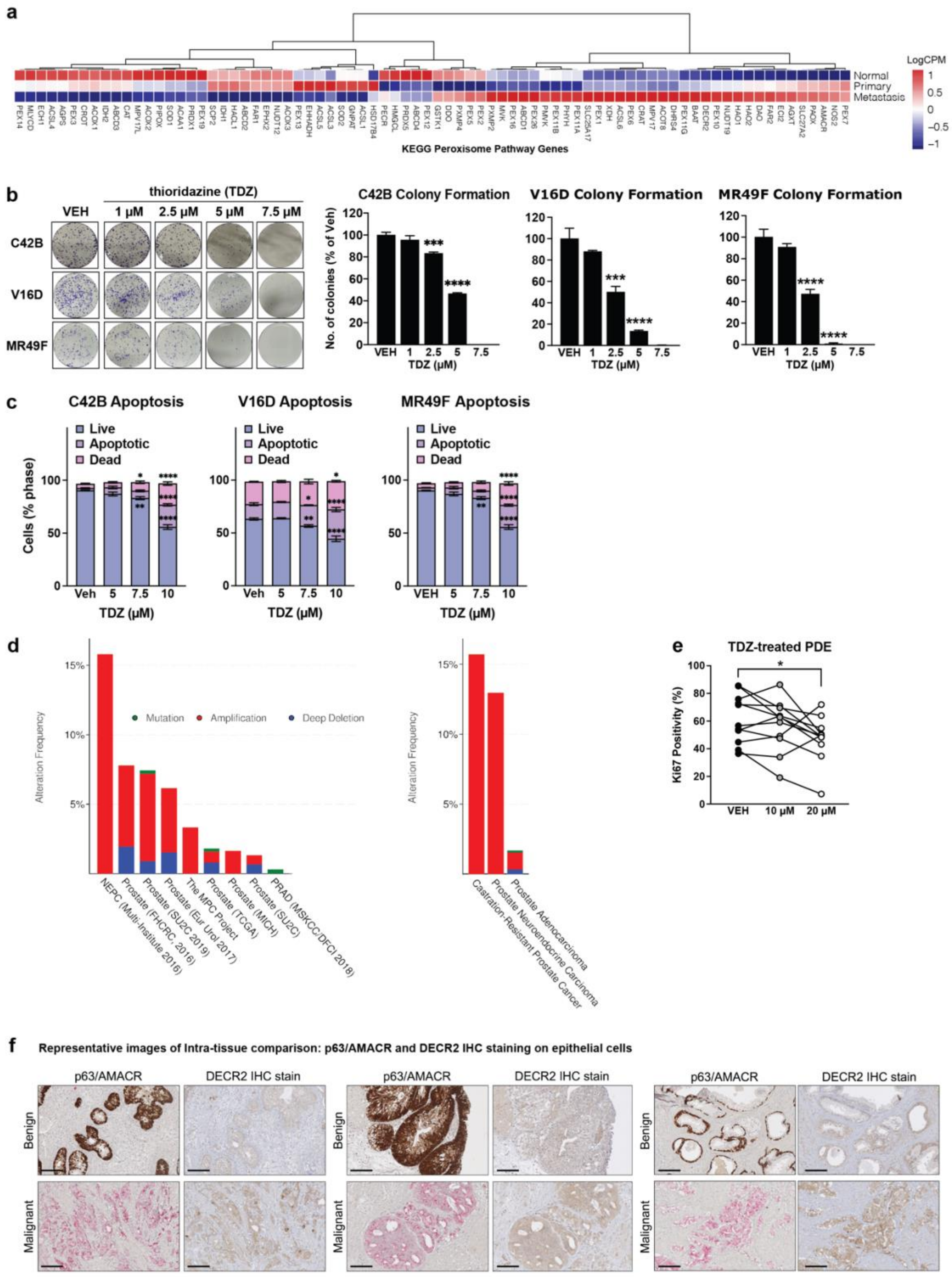

Supplementary Figure 1

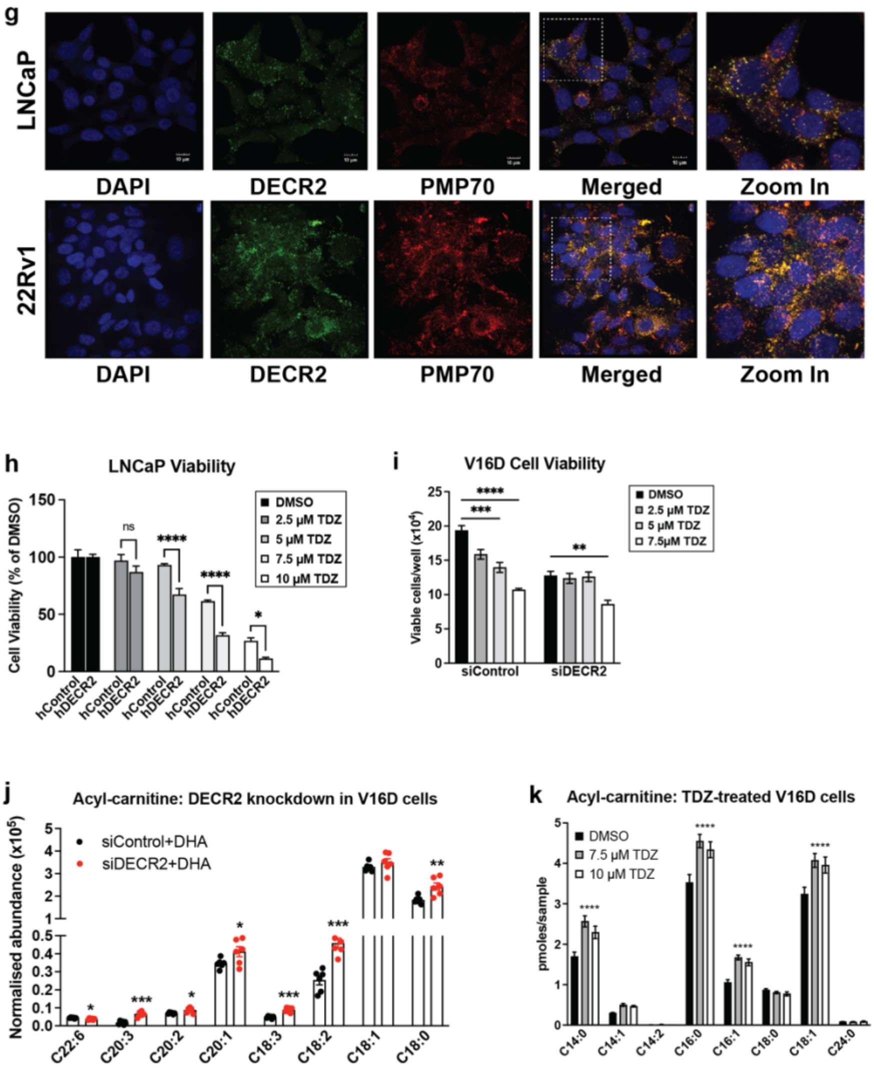

Supplementary Figure 2

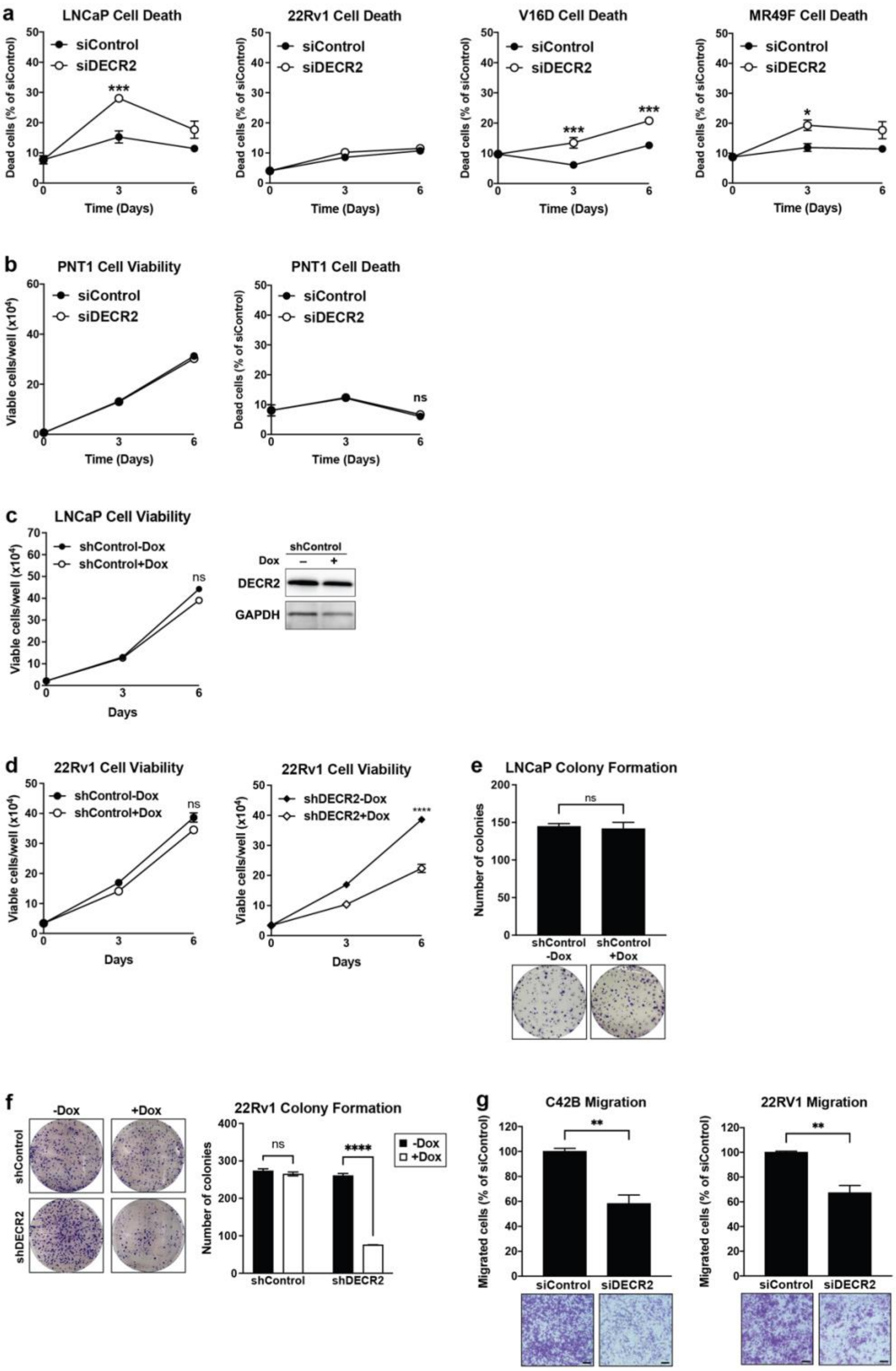

Supplementary Figure 2

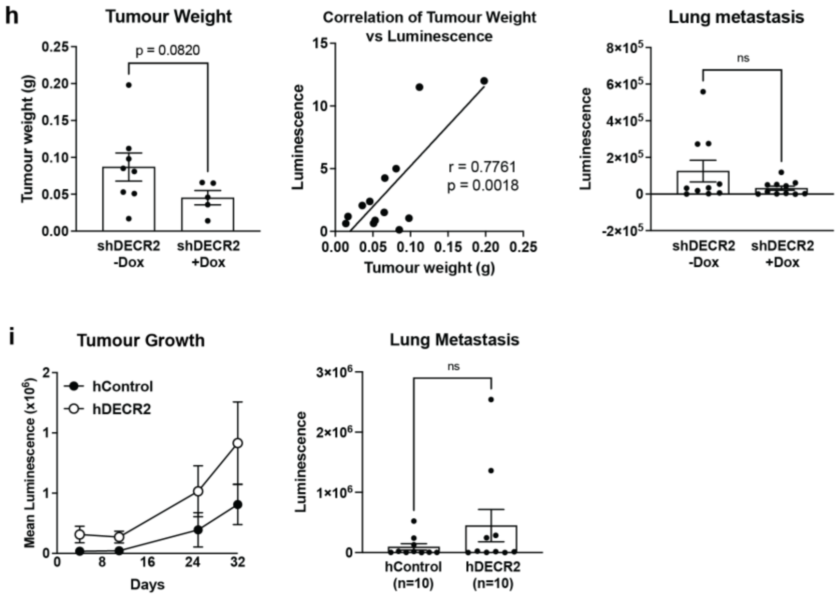

Supplementary Figure 3

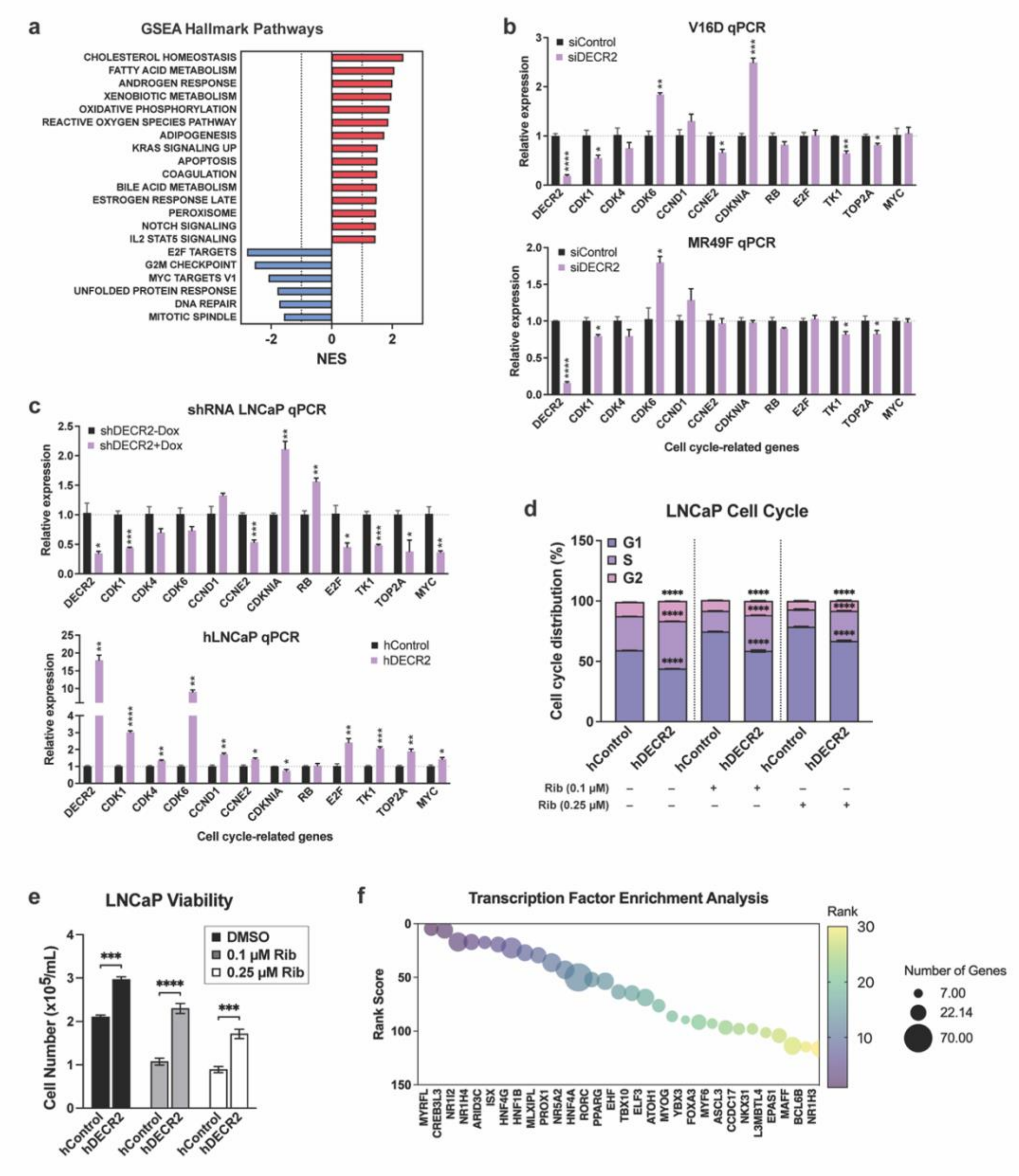

Supplementary Figure 4

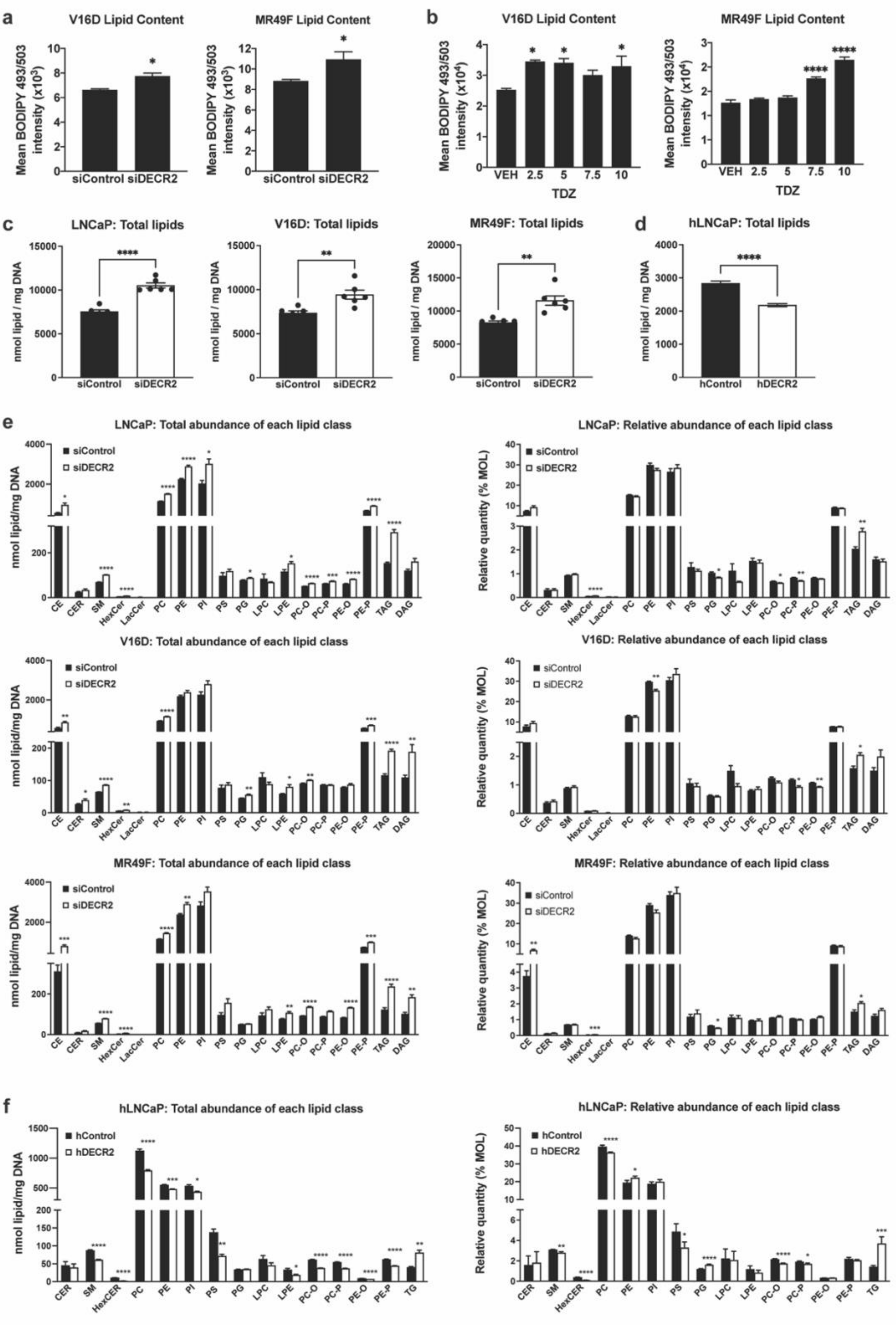

104 **Supplementary Figure 5**

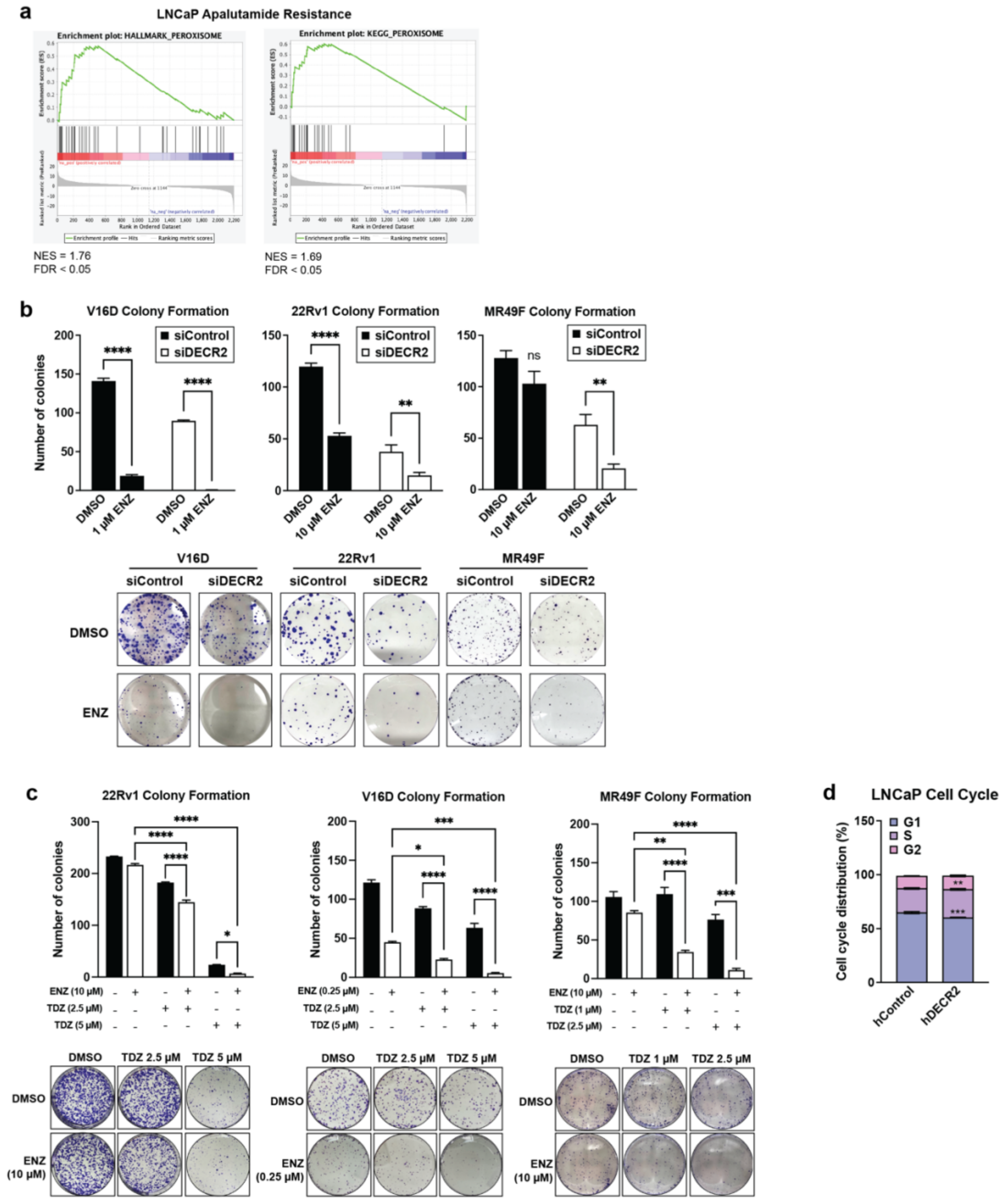

Table 1 The clinicopathologic features of prostate cancer patients included in this study

Patient ID	Age at RP	Pre-OP PSA	Gleason Score (Pre-OP)	1° GG	2° GG	3° GG	Pathological Staging
33975R	65.3	5	7	4	3		PT3A
33976LA	82.5	7.6	7	4	3		PT2
33985L	61.2	5.3	7	3	4		PT3A
33986L	73.6	7.9	7	4	3		PT3A
33987L	70.5	9.14	6	3	3		PT3A
33988RB	70.1	5.17	7	3	4		PT2
33989RA	69.1	6.6	7	4	3		PT2
33992L	74	6.16	7	3	4		PT2
33993L	70.5	10.5	6	3	3		PT2
33994R	58.2	8.75	8	4	4	3	PT2

Table 2 List of primary antibodies

Antibody	Conjugate	Species	Identifier	Supplier
β-actin	None	Mouse	A5441	Sigma-Aldrich
HSP90	None	Rabbit	4874S	Cell Signalling Technology
DECR2	None	Rabbit	ab153849	Abcam
AR	None	Rabbit	sc-816	Santa Cruz
PMP70	None	Mouse	SAB4200181	Sigma-Aldrich
Rb	None	Mouse	9309	Cell Signalling Technology
pRb	None	Rabbit	ab184796	Abcam
p21	None	Rabbit	SC-317	Santa Cruz
p27	None	Rabbit	SC-528	Santa Cruz
cyclin D1	None	Rabbit	M3642	DAKO
CDK4	None	Rabbit	SC-260	Santa Cruz
GAPDH	Rhodamine	Rabbit	12004167	BioRad
Ki67	Mouse	M7240		DAKO
Rabbit	HRP	Rabbit		DAKO
Mouse	HRP	Mouse		DAKO

Table 3 Primer sequences

Gene	Primer (F/R)	Sequence
GUSB	GUSB-F	CGTCCCACCTAGAATCTGCT
	GUSB-R	TTGCTCACAAAGGTCACAGG
L19	L19-F	TGCCAGTGGAAAAATCAGCCA
	L19-R	CAAAGCAAATCTCGACACCTTG
DECR2	DECR2-F	TACCGCCACCTCTTCTGC
	DECR2-R	CTCCTACTGGCAATCACCGT
CDK1	CDK1-F	TTGGATTCTATCCCTCCTGGT
	CDK1-R	ACAATCCCCTGTAGGATTTGG
CDK4	CDK4-F	CCGAAGTTCTTCTGCAGTCC
	CDK4-R	GTCGGCTTCAGAGTTTCCAC
CDK6	CDK6-F	TGGAGACCTTCGAGCACC
	CDK6-R	CACTCCAGGCTCTGGAACTT
CCND1	CCND1-F	CAGAGGCGGAGGAGAACAAA
	CCND1-R	AGGGCGGATTGGAAATGAACT
CCNE2	CCNE2-F	ACCTCATTATTCATTGCTTCCAA
	CCNE2-R	TCTTCACTGCAAGCACCATC
CDKN1A	CDKN1A-F	GACTCTCAGGGTCGAAAACG
	CDKN1A-R	GGATTAGGGCTTCCTCTTGG
RB1	RB1-F	CAGAAGGCAACTTGACAAGAGA
	RB1-R	CCTTCTCGGTCCTTTGATTG
E2F	E2F-F	CATCCCAGGAGGTCACTTCT
	E2F-R	GACAACAGCGGTTCTTGCTC
TK1	TK1-F	CTGTCATAGGCATCGACGAG
	TK1-R	TCCAGTGCAGCCACAATTAC
TOP2A	TOP2A-F	TGAAGGAAGCCCTCAAGAAG
	TOP2A-R	TGGCTTAAATGCCAATGTAGTTT
MYC	MYC-F	AGCGACTCTGAGGAGGAACA
	MYC-R	CTCTGACCTTTTGCCAGGAG

Table 4 DECR2 shRNA and overexpression sequences

Expression target	Sequence
**Human DECR2**<br>shRNA	mature antisense: ACAAGTCTCGGGATCCATG
**Human DECR2**:<br>subcloned human<br>target coding sequence<br>(GenTarget Inc)	atggcccagccgccgcccgacgtggagggggacgactgtctccccgcgtaccgcc<br>acctcttctgcccggacctgctgcgggacaaagtggccttcatcacaggaggcggct<br>ctgggattgggttccggattgctgagattttcatgcggcacggctgccatacggtgattg<br>ccagtaggagcctgccgcgagtgctgacggccgccaggaagctggctggggccac<br>cggccggcgctgcctccctctctctatggacgtccgagcgcccccagctgtcatggcc<br>gccgtggaccaggctctgaaggagtttggcagaatcgacattctcattaactgtgcgg<br>ccgggaacttcctgtgccccgctggcgccttgtccttcaacgccttcaagaccgtgatg<br>gacatcgataccagcggcaccttcaatgtgtctcgtgtgctctatgagaagttcttccgg<br>gaccacggaggggtgatcgtgaacatcactgccaccctggggaaccgggggcag<br>gcgctccaggtgcatgcaggctccgccaaggccgctgtggacgcgatgacgcggc<br>acttggctgtggagtggggtccccaaaacatccgcgtcaacagcctcgcccctggcc<br>ccatcagtggcacagaggggctccggcgactgggtggccctcaggccagcctgag<br>caccaaggtcactgccagcccgctgcagaggctggggaacaagaccgagatcgc<br>ccacagcgtgctctacctggccagccctctggcttcctacgtgacgggggccgtgctg<br>gtggccgatggcggggcatggttgacgttcccaaacggtgtcaaagggctgccggat<br>ttcgcatccttctctgctaagctc
